# Therapeutic Enhancement of Protective Immunity during Experimental Leishmaniasis

**DOI:** 10.1371/journal.pntd.0001316

**Published:** 2011-09-06

**Authors:** Senad Divanovic, Aurelien Trompette, Jamie I. Ashworth, Marepalli B. Rao, Christopher L. Karp

**Affiliations:** 1 Division of Molecular Immunology, Cincinnati Children's Hospital Medical Center, and the University of Cincinnati College of Medicine, Cincinnati, Ohio, United States of America; 2 Department of Environmental Health, University of Cincinnati College of Medicine, Cincinnati, Ohio, United States of America; Yale School of Public Health, United States of America

## Abstract

**Background:**

Leishmaniasis remains a significant cause of morbidity and mortality in the tropics. Available therapies are problematic due to toxicity, treatment duration and emerging drug resistance. Mouse models of leishmaniasis have demonstrated that disease outcome depends critically on the balance between effector and regulatory CD4^+^ T cell responses, something mirrored in descriptive studies of human disease. Recombinant IL-2/diphtheria toxin fusion protein (rIL-2/DTx), a drug that is FDA-approved for the treatment of cutaneous T cell lymphoma, has been reported to deplete regulatory CD4^+^ T cells.

**Methodology/Principal Findings:**

We investigated the potential efficacy of rIL-2/DTx as adjunctive therapy for experimental infection with *Leishmania major*. Treatment with rIL-2/DTx suppressed lesional regulatory T cell numbers and was associated with significantly increased antigen-specific IFN-γ production, enhanced lesion resolution and decreased parasite burden. Combined administration of rIL-2/DTx and sodium stibogluconate had additive biological and therapeutic effects, allowing for reduced duration or dose of sodium stibogluconate therapy.

**Conclusions/Significance:**

These data suggest that pharmacological suppression of immune counterregulation using a commercially available drug originally developed for cancer therapy may have practical therapeutic utility in leishmaniasis. Rational reinvestigation of the efficacy of drugs approved for other indications in experimental models of neglected tropical diseases has promise in providing new candidates to the drug discovery pipeline.

## Author Summary

Leishmaniasis is an infectious disease that causes a large burden of morbidity and mortality in the tropics. Caused by protozoan parasites of the genus *Leishmania* that are transmitted by sandflies, leishmaniasis causes a wide spectrum of human disease. The severe end of the spectrum, visceral leishmaniasis, causes an annual mortality of approximately 50,000, largely in India and Sudan. Available therapies for leishmaniasis are problematic due to emerging drug resistance, toxicity and/or the need for lengthy courses of treatment. There is thus an urgent need for novel therapeutic approaches to this neglected tropical disease. To address this problem, the authors examined whether a commercially available drug developed for cancer therapy (Ontak), reported to have immunological activity of relevance to the immunobiology of *Leishmania* infection, exhibited efficacy in mouse models of leishmaniasis. The study found therapeutic efficacy for the drug alone in these models, as well as additive therapeutic efficacy in combination with standard antimicrobial therapy. Rational reinvestigation of the efficacy of already approved drugs in experimental models of neglected tropical diseases has promise in providing needed new candidates to the drug discovery pipeline.

## Introduction

Protozoa of the genus *Leishmania* cause a wide spectrum of human disease [1]. At the severe end of the spectrum, visceral leishmaniasis (kala azar), due to disseminated parasitism of macrophages and dendritic cells, causes an annual mortality of approximately 50,000, largely in India and Sudan [2]. Kala azar has also emerged as a significant problem in the setting of HIV/AIDS, visceral leishmaniasis being the second most common opportunistic tissue protozoal disease (after toxoplasmosis) in people infected with HIV [3]. Available therapies for kala azar, including pentavalent antimonials, some (but not all [4]) amphotericin B preparations, miltefosine and paromomycin, are problematic due to emerging drug resistance, toxicity, need for lengthy treatment and/or the development of post-kala azar dermal lesions [5], [6], [7], [8], [9]. There is thus a clear need for novel therapeutic approaches to this neglected tropical disease.

Experimental mouse models of *Leishmania* infection have been used extensively to interrogate the immune system as well as the immunopathogenesis of leishmaniasis [10], [11], [12], [13]. Inoculation of low numbers of *L. major* into the dermis of C57BL/6 mice is followed by the recruitment of antigen-specific effector CD4^+^ and CD8^+^ T cells, IFN-γ production at the site of infection, and activation of the microbicidal effector functions of parasitized macrophages, events manifested clinically by lesion development [13]. IL-10 production by CD4^+^ T cells is critical to immune counterregulation in this model. Balanced IFN-γ and IL-10 responses are essential for disease resolution and the establishment of life-long latent infection [14]. IFN-γ deficiency or neutralization leads to systemic parasite spread [15], [16]; IL-10 deficiency or neutralization leads to sterile cure [17], [18]. A similar balance between IFN-γ and IL-10 responses also appears to be a critical determinant of the outcome of human leishmaniasis [19]. Several relevant IL-10-producing CD4^+^ T cell subsets have been described, including natural and adaptive regulatory T cells (T_reg_) and Th_1_ cells that produce IL-10 in addition to IFN-γ [20], [21], [22]. Recent studies have emphasized the role played by the latter cells in immune counterregulation in experimental leishmaniasis [20], [23] and human visceral leishmaniasis [24]. That said, monoclonal antibody-mediated depletion of CD25 (IL-2R)-expressing cells, a technique that depletes T_reg_ cells, has been reported to facilitate parasite eradication in experimental leishmaniasis, in models of primary infection and superinfection, as well as in vaccination models [25], [26], [27], [28].

Denileukin diftitox (rIL-2/diphtheria toxin [DTx]), a recombinant fusion protein composed of the membrane-translocating and cytotoxic domains of diphtheria toxin (Met1-Thr387)-His and human interleukin 2 (Ala1-Thr133), is FDA-approved for the treatment of cutaneous T cell lymphoma [29]. Internalization of rIL-2/DTx into cells expressing the high affinity IL-2 receptor leads to activation of the ADP-ribosyltransferase function of DTx in the endosome. Activated DTx is subsequently translocated into the cytosol where it inhibits protein synthesis and induces apoptosis [29]. rIL-2/DTx treatment leads to a significant reduction in peripheral blood CD4^+^CD25^+^Foxp3^+^ T_reg_ populations in humans [30]. Furthermore, clinical treatment of patients with rIL-2/DTx has been reported to enhance immune responses [30], [31], [32]. Similarly, treatment of mice with rIL-2/DTx has been reported to decrease splenic, bone marrow and peripheral blood CD4^+^CD25^+^Foxp3^+^ T_reg_ [33]. Such treatment has been shown to have benefit in experimental tumor models [34] and several experimental models of immune-mediated disease [35], [36], [37], [38], [39].

Given these data, we hypothesized that rIL-2/DTx treatment would enhance the resolution of experimental *L. major* infection. Treatment with rIL-2/DTx reduced T_reg_/CD4^+^ T cell ratios during experimental *L. major* infection, increasing antigen-specific IFN-γ production, enhancing lesion resolution and decreasing parasite burden. Furthermore, combined administration of rIL-2/DTx and sodium stibogluconate had additive biological and therapeutic effects, in both genetically resistant (C57BL/6) and susceptible (BALB/c) mice.

## Methods

### Mice

Female C57BL/6 and BALB/c mice were purchased from Jackson Laboratories. All animals were housed in a specific pathogen-free animal facility, in high-efficiency particulate-filtered laminar flow hoods with free access to food and water, at Cincinnati Children's Hospital Medical Center (CCHMC). Animal care was provided in accordance with the procedures outlined in the Guide for the Care and Use of Laboratory Animals under animal study proposals approved by the CCHMC IACUC.

### *In vivo* infection model

*L. major* clone V1 (MHOM/IL/80/Friedlin) promastigotes were grown at 28°C in medium 199 (Cellgro), supplemented with 20% fetal calf serum (FCS) [Hyclone], 100 U/ml penicillin and 100 µg/ml streptomycin (Cellgro), 25 mM HEPES (Invitrogen), 2 mM L-glutamine, 0.1 mM adenine, 5 µg/ml hemin, and 2 µg/ml d-biotin (all from Sigma), and passaged at least 3 times, but not more than 5 times, prior to infection. Ficoll gradient purification [40] was used to purify infectious phase metacyclic promastigotes from 5 d old stationary cultures. 8 week-old mice were infected in the dermis of the ear with 3×10^3^ *L. major* metacyclic promastigotes in 10 µl FBS-free media. Lesion size was quantified with vernier calipers. All reagents used for *in vivo* infection were endotoxin-free to the limits of detection of the *Limulus* amebocyte lysate assay (Bio-Whittaker).

Mice were treated intraperitoneally with rIL-2/DTx (Denileukin diftitox, ONTAK; Ligand Pharmaceuticals, Inc.), intramuscularly with sodium stibogluconate (SSG; The Wellcome Foundation, Inc., provided by the Centers for Disease Control and Prevention), and/or an equal volume of sterile, endotoxin-free saline (Hospira Inc.) via these same routes as a control.

### Parasite quantification

To quantify lesional parasite burden, the ventral and dorsal sheets of the infected ears were separated, deposited dermal side down into 24-well tissue culture plates containing RPMI (Cellgro) supplemented with 100 U/ml penicillin, 100 µg/ml streptomycin and 50 µg/ml liberase CI enzyme blend (Roche), and incubated for 45 min at 37°C. Tissues were subsequently dissociated in RPMI containing 10% FCS and 0.05% DNAse I (Sigma) using a medimachine (BD Biosciences), according to the manufacturer's protocol. Tissue homogenates were filtered using a 50 µm cell strainer (Falcon Products Inc) and serially diluted (1∶2) in 96-well flat bottom microtiter tissue culture plates containing 50 µl of Novy-MacNeal-Nicolle (NNN) medium with 20% defibrinated rabbit blood (Hemostat Laboratories) overlaid with 100 µl medium 199 supplemented with 20% FCS, 100 U/ml penicillin, 100 µg/ml streptomycin, 2 mM L-glutamine, 25 mM HEPES, 0.1 mM adenine, 5 µg/ml hemin, and 2 µg/ml d-biotin. After culture for 7 d at 28°C the number of viable parasites was quantified by limiting dilution analysis. The parasite burden in draining, retromaxillar lymph nodes, liver (left lobe) and spleen was quantified by limiting dilution analysis using similar procedures. All reagents used for cell culture and parasite titration were endotoxin-free to the limits of detection of the *Limulus* amebocyte lysate assay (Bio-Whittaker).

### Flow cytometric analysis

Single cell suspensions generated from lesional sites or draining lymph nodes, obtained as described above, were treated with FACS fix buffer for 15 min (BD Biosciences). Cells were washed and co-incubated with anti-FcγIII/II (CD16/32; e-Bioscience) antibody for 30 min in PBS containing 0.1% BSA and 0.01% sodium azide. After a further wash, cells were incubated with directly-conjugated monoclonal antibodies to TCR-β-FITC (H57-597), CD4-PE-Cy7 (RM4-5) or CD4-APC-Alexa Fluorochrome 750 (RM4-5), CD8-Pacific Blue (53-6.7), CD25-PE (PC61), NK1.1-PerCp-Cy5.5 (PK136), CD49b-PE-Cy7 (DX5), F4/80-APC (BM8), CD11b-PerCp-Cy5.5 (M1/70), Gr-1-FITC (RB6-8C5), CD11c-Alexa Fluorochrome 700 (N418), B220-APC-Alexa Fluorochrome 750 (RA3-6B2), and/or CD19-PE (1D3) [all antibodies were from BD Biosciences and/or e-Bioscience] for 30 min.

Quantification of Foxp3 expression was done using the Foxp3-APC (FJK-16s) staining kit (e-Bioscience) according to manufacturer's instructions, combined with directly-conjugated monoclonal antibodies TCR-β-FITC, CD4-APC-Alexa Fluorochrome 750 and CD25-PE (BD Biosciences and/or e-Bioscience).

Isotype control antibodies (BD Biosciences and/or e-Bioscience) were used in each analysis. Data were collected and analyzed using a combination of FACSCalibur flow cytometer and CellQuest software or LSRII flow cytometer and FACSDiva software (BD Immunocytometry Systems).

### Antibody detection

96-well, EIA/RIA flat-bottom, plates (Costar) were coated with diphtheria toxin (1 µg/ml; Sigma) in 50 mM carbonate/bicarbonate buffer pH 9.6 and incubated overnight at 4°C. Plates were washed (6×) with wash buffer (Tris-Buffer Saline pH 7.2 and 0.05% Tween 20), serum samples, diluted in dilution buffer (wash buffer supplemented with 10% SuperBlock [Pierce]), were added and incubated for 30 min at room temperature. Plates were washed, alkaline phosphatase-conjugated anti-mouse IgG1 antibody (1∶1000 in dilution buffer; BD Biosciences) was added and plates were incubated for an additional 30 min at room temperature. After a further wash, pNPP (1 mg/ml; Calbiochem) in TM Buffer (Tris Base supplemented with 0.3 M MgCl_2_, pH 9.8) was added and optical density (405 nm) was quantified using kinetic microplate reader (Molecular Devices).

### Cytokine secretion

Draining lymph node cells were plated in 96-well tissue culture plates at 5×10^6^ cells/ml and cultured for 96 h at 37°C in 5% CO_2_ in RPMI containing 100 U/ml penicillin, 100 µg/ml streptomycin, 10% fetal calf serum, 0.1 mM β-mercaptoethanol (Invitrogen) and soluble *L. major* antigens were generated as described [41]. Secreted IFN-γ, IL-10 and IL-4 were quantified by ELISA (BD Biosciences).

### Statistical analysis

Kinetic lesion size data was first analyzed by MANOVA, to reject the null hypothesis of equal effects, followed by ANOVA (plus Tukey's multiple comparison test) or the unpaired Student's *t* test, as appropriate. In studies aimed at defining whether rIL-2/DTx treatment allowed for a reduction in the dose or duration of standard antimicrobial therapy, linear random effects (time) modeling was done to test the null hypothesis that all treatments had equal effects, as well as to sort the therapeutic efficacy of the diverse treatment regimens. Parasite numbers were log-transformed before analysis, and analyzed by ANOVA (followed by Tukey's multiple comparison test) or the unpaired Student's *t* test, or the non-parametric Kruskal-Wallis test (followed by the Wilcoxon test), as appropriate.

## Results

### Kinetics of *in vivo* T_reg_ depletion by rIL-2/DTx

**Figure 1:**
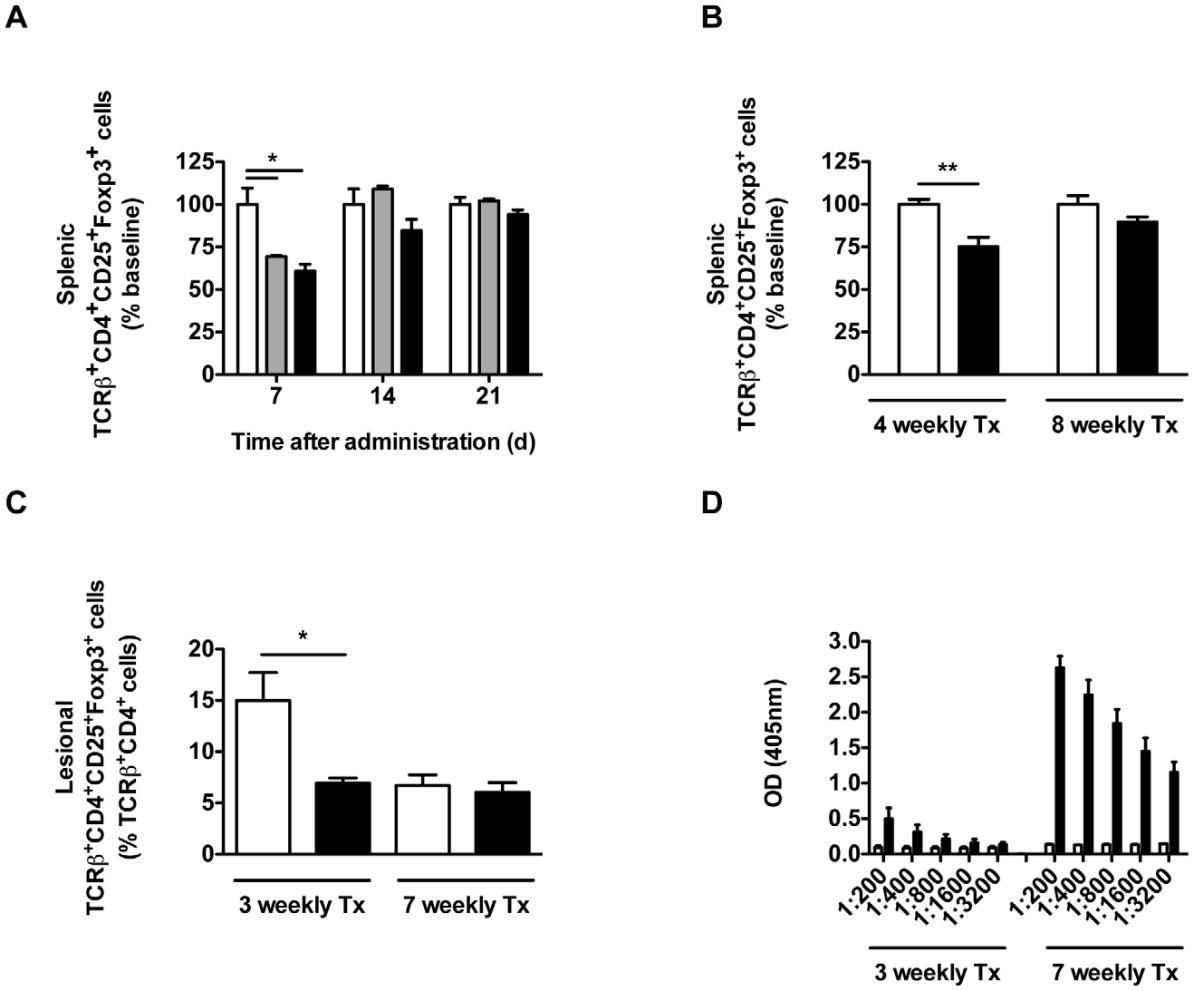
Short-term treatment with rIL-2/DTx leads to transient T_reg_ depletion. (**A**) Uninfected C57BL/6 mice were treated intraperitoneally with a single dose of normal saline (open bars) or rIL-12/DTx (12 µg/kg, gray bars; 50 µg/kg, black bars) and splenic T_reg_ (TCRβ^+^CD4^+^CD25^+^Foxp3^+^ cells) were quantified by flow cytometry at the time indicated. (**B**) Uninfected C57BL/6 mice were treated intraperitoneally with 4 or 8 weekly doses of normal saline (open bars) or rIL-12/DTx 50 µg/kg (filled bars) and splenic T_reg_ (TCRβ^+^CD4^+^CD25^+^Foxp3^+^ cells) were quantified by flow cytometry 7 d after the final dose. (**C** and **D**) C57BL/6 mice were given weekly intraperitoneal doses of normal saline (open bars) or rIL-2/DTx (50 µg/kg; filled bars), starting 1 week after intradermal infection in both ears with 3×10^3^ metacyclic *L. major* promastigotes. Lesional T_reg_ (TCRβ^+^CD4^+^CD25^+^Foxp3^+^ cells) were quantified by flow cytometry (**C**), and IgG_1_ antibodies to diphtheria toxin were measured by ELISA in serially diluted serum samples (**D**), 7 d after the last indicated dose of rIL-2/DTx. Data represent means +/− SE in a single experiment; *n* = 3 (**A**), *n* = 4–6 (**B**) and *n* = 5–6 (**C** and **D**). (**A**) ANOVA *P*<0.01; Tukey's correction; *P<0.05; (**B** and **C**) Student's *t* test *P<0.05, **P<0.01.

Given the robust expression of the high affinity IL-2 receptor by Foxp3-expressing T_reg_, it is not surprising that rIL-2/DTx treatment has been reported to deplete T_reg_ in humans and mice [30], [31], [33], [34], [42], [43]. To define the kinetics of rIL-2/DTx-mediated T_reg_ depletion, we treated uninfected mice with a single injection of rIL-2/DTx (or vehicle control) and quantified splenic T_reg_ numbers thereafter. As shown in Figure 1A, rIL-2/DTx injection led to a significant decrease in the percentage of splenic T_reg_ quantified one week after treatment (see Fig. S1 for flow cytometric gating strategy). However this reduction was not sustained; no alterations in T_reg_ percentage were observed two or three weeks after administration of a single dose (Figure 1A). A similar significant reduction in the percentage of splenic T_reg_ was observed after four weekly doses of rIL-2/DTx (Figure 1B). However, longer treatment (8 weekly doses) failed to result in sustained T_reg_ depletion (Figure 1B). During the course of experimental cutaneous leishmaniasis, a dynamic process of T_reg_ recruitment to and retention in lesional sites has been observed [41]. Short-term administration (3 weekly doses) of rIL-2/DTx, 1 week after *L. major* infection, resulted in a significant reduction in lesional, draining lymph node and splenic T_reg_ accumulation after 4 weeks of infection (Figure 1C and data not shown). However, similar to findings in the spleens of uninfected mice, prolonged administration of rIL-2/DTx (7 weekly doses), 1 week after *L. major* infection, failed to result in sustained lesional T_reg_ depletion; lesional T_reg_ percentages were similar in treated and mock-treated mice 8 weeks after infection (Figure 1C). It will be noted that, with lesional healing in these genetically resistant mice, lesional T_reg_ numbers decrease in untreated mice over this time frame as well. As shown in Figure 1D, such prolonged administration of rIL-2/DTx led to the generation of robust titers of anti-DTx antibodies.

### Short-term administration of rIL-2/DTx enhances lesion resolution and reduces *L. major* parasite burden

**Figure 2:**
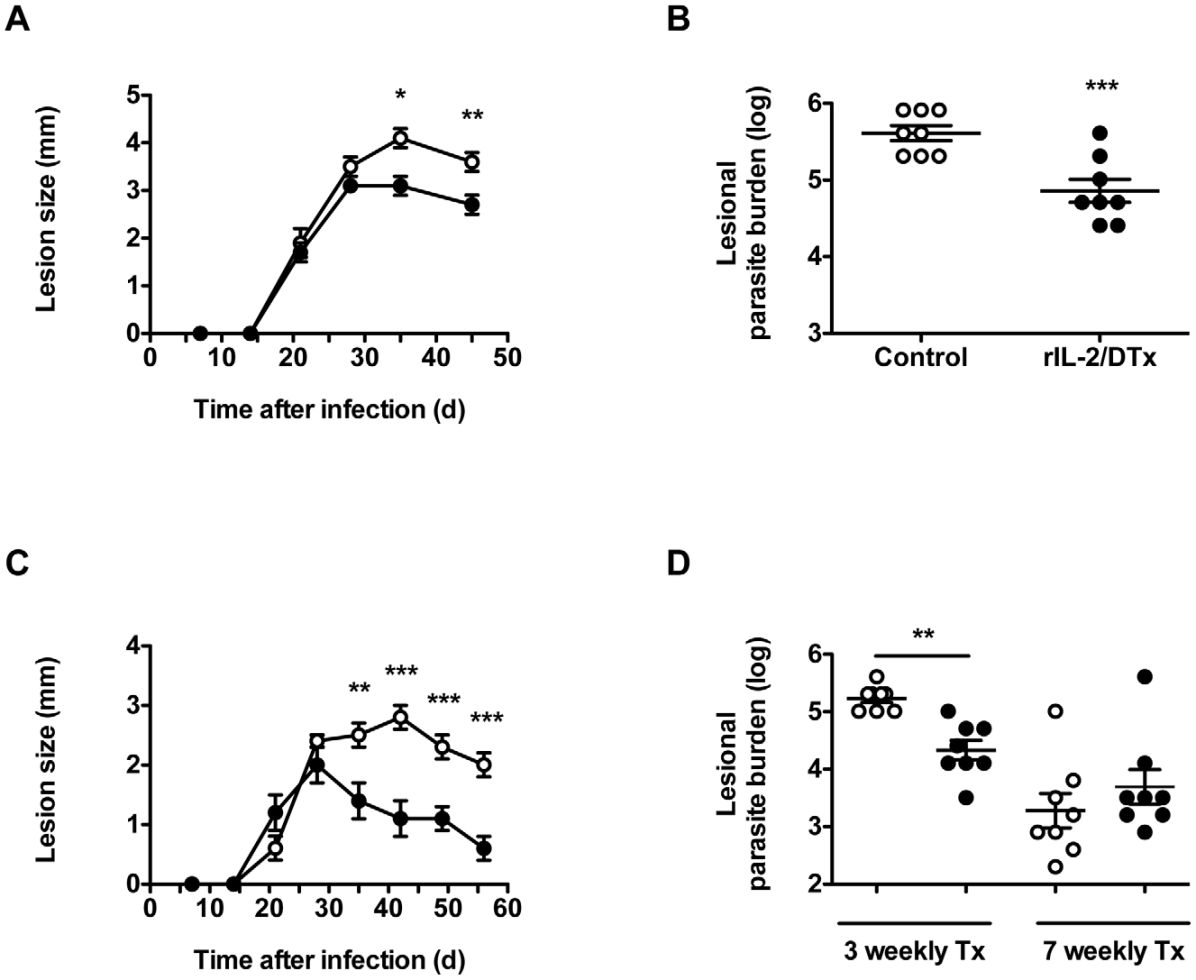
Treatment with rIL-2/DTx enhances resolution of experimental *L. major* infection. C57BL/6 mice were infected intradermally in both ears with 3×10^3^ metacyclic *L. major* promastigotes. (**A**, **B**) Beginning 30 d after infection, mice were treated three times, at 5 d intervals, with normal saline (open symbols) or rIL-2/DTx (12 µg/kg; filled symbols). (**A**) Lesion size; (**B**) Lesional parasite burden, quantified 45 d after infection. (**C**, **D**) Beginning 7 d after infection, mice were treated at weekly intervals with normal saline (open symbols) or rIL-2/DTx (12 µg/kg; filled symbols), and lesion size (**C**) and parasite burden (**D**) was quantified 7 d after administration of the last indicated dose. Data represent means +/− SE of 8 mice/group (with individual data points shown for parasite burden). (**A** and **C**) MANOVA *P*<0.05; (**A–D**) Student's *t* test; *P<0.01, **P<0.005, ***P<0.001.

To define the effectiveness of rIL-2/DTx administration on the resolution of an ongoing *L. major* infection, mice were treated with rIL-2/DTx, or vehicle as a control, beginning 30 d after infection. In light of the above kinetic data, the mice were given three doses of drug or vehicle, at 5 d intervals. As shown in Figure 2, rIL-2/DTx treatment significantly enhanced lesion resolution (Figure 2A) and resulted in a significant decrease in lesional parasite burden (Figure 2B).

While not especially relevant to therapy of human disease, we also examined the effect of weekly therapy with rIL-2/DTx, beginning 1 week after infection, on experimental cutaneous leishmaniasis. This protocol also significantly enhanced lesion resolution compared to control therapy (Figure 2C)— something sustained from the onset of lesion resolution in rIL-2/DTx-treated mice through the rest of the 8-week time course of the experiment. However, while such therapy led to significantly decreased lesional parasite burden after 3 weekly doses of rIL-2/DTx (Figure 2D), no significant enhancement (or impairment) of host control of parasite burden was observed after 7 weekly doses of rIL-2/DTx therapy, compared with mock therapy (Figure 2D), something perhaps predictable both from the generation of antibodies to DTx observed with this protocol (Figure 1D), as well as the baseline levels of host resistance observed in this model.

### rIL-2/DTx and sodium stibogluconate have additive therapeutic efficacy against experimental *L. major* infection

**Figure 3:**
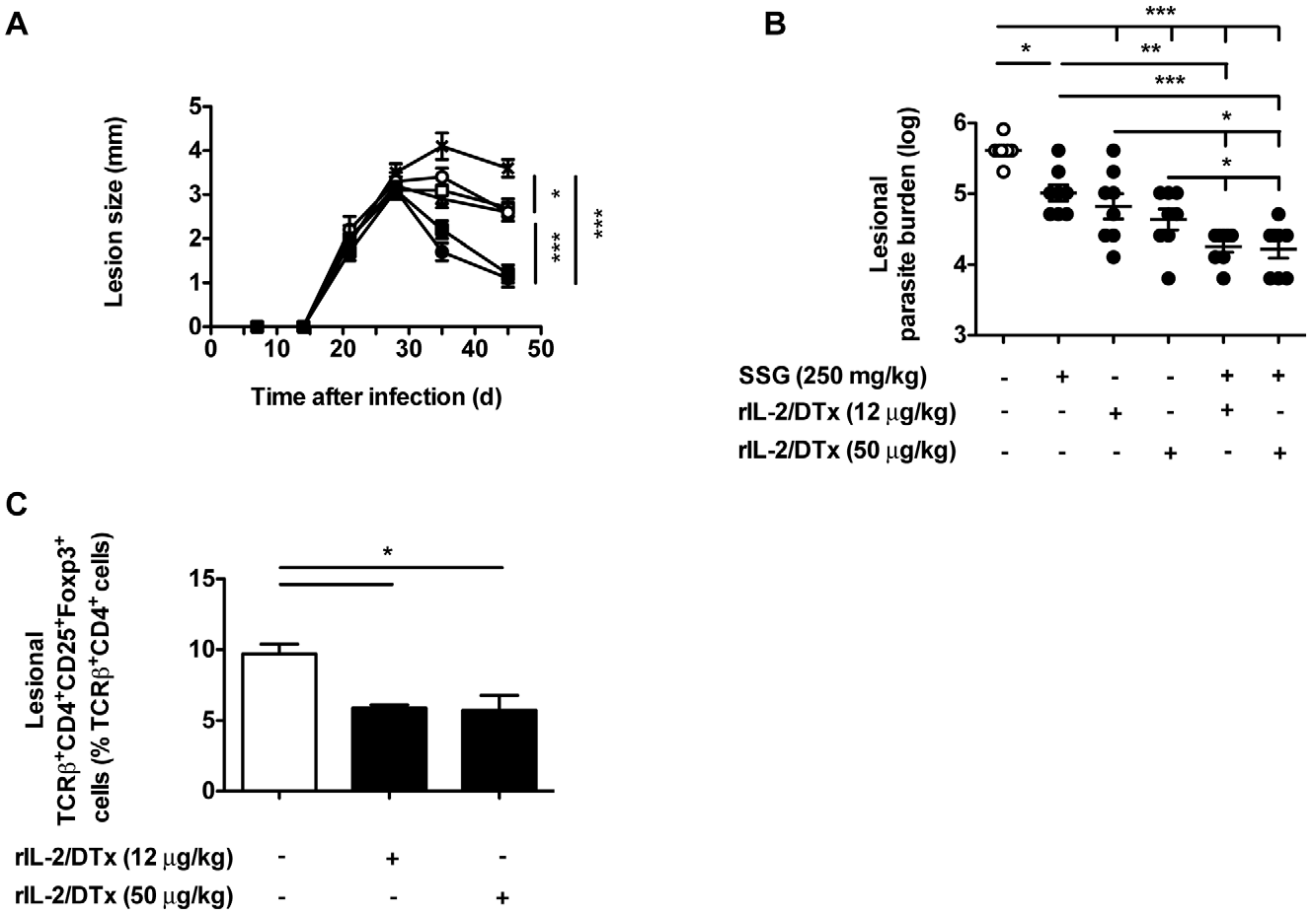
Combined rIL-2/DTx and sodium stibogluconate treatment provides additive efficacy in experimental *L. major* infection. C57BL/6 mice were infected as in Figure 2. Beginning 30 d after infection, mice were treated: (1) daily for 10 d with SSG (250 mg/kg; filled triangles); (2) three times at 5 d intervals with rIL-2/DTx (12 µg/kg; open squares); (3) three times at 5 d intervals with rIL-2/DTx (50 µg/kg; open circles); (4) daily for 10 d with SSG (250 mg/kg) plus three times at 5 d intervals with rIL-2/DTx (12 µg/kg; filled squares); or (5) daily for 10 d with SSG (250 mg/kg) plus three times at 5 d intervals with rIL-2/DTx (50 µg/kg; filled circles); or (6) with normal saline (per route and schedule for 10 d SSG plus 3 doses of rIL-2/DTx; crossed symbols). (**A**) Lesion size, (**B**) lesional parasite burden, and (**C**) lesional T_reg_ percentage were quantified in the indicated groups, 45 d after infection. Data represent means +/− SE of 8 mice/group in a single experiment (with individual data points shown for parasite burden); *n* = 8 (**A** and **B**), *n* = 4 (**C**). (**A**) MANOVA *P*<0.05; (**A–C**) ANOVA *P*<0.02; Tukey's correction; *P<0.05, **P<0.01, ***P<0.001.

We next examined the therapeutic effect of co-administration of rIL-2/DTx and pentavalent antimony (sodium stibogluconate [SSG]) on the resolution of *L. major* infection, again, beginning therapy 30 d after infection. Single agent therapy with either rIL-2/DTx or SSG led to significant improvement in lesion resolution and significant decreased parasite burden, compared to vehicle-treated animals (Figure 3A and 3B). Further, combined therapy with rIL-2/DTx and SSG, regardless of the dose of rIL-2/DTx employed, resulted in significantly enhanced lesion resolution and decreased parasite burden compared to single agent therapy (Figure 3A and 3B). As expected, rIL-2/DTx treatment led to a significant reduction in lesional T_reg_ (Figure 3C). The addition of SSG to rIL-2/DTx (or vehicle) treatment failed to alter lesional T_reg_ (data not shown).

**Figure 4:**
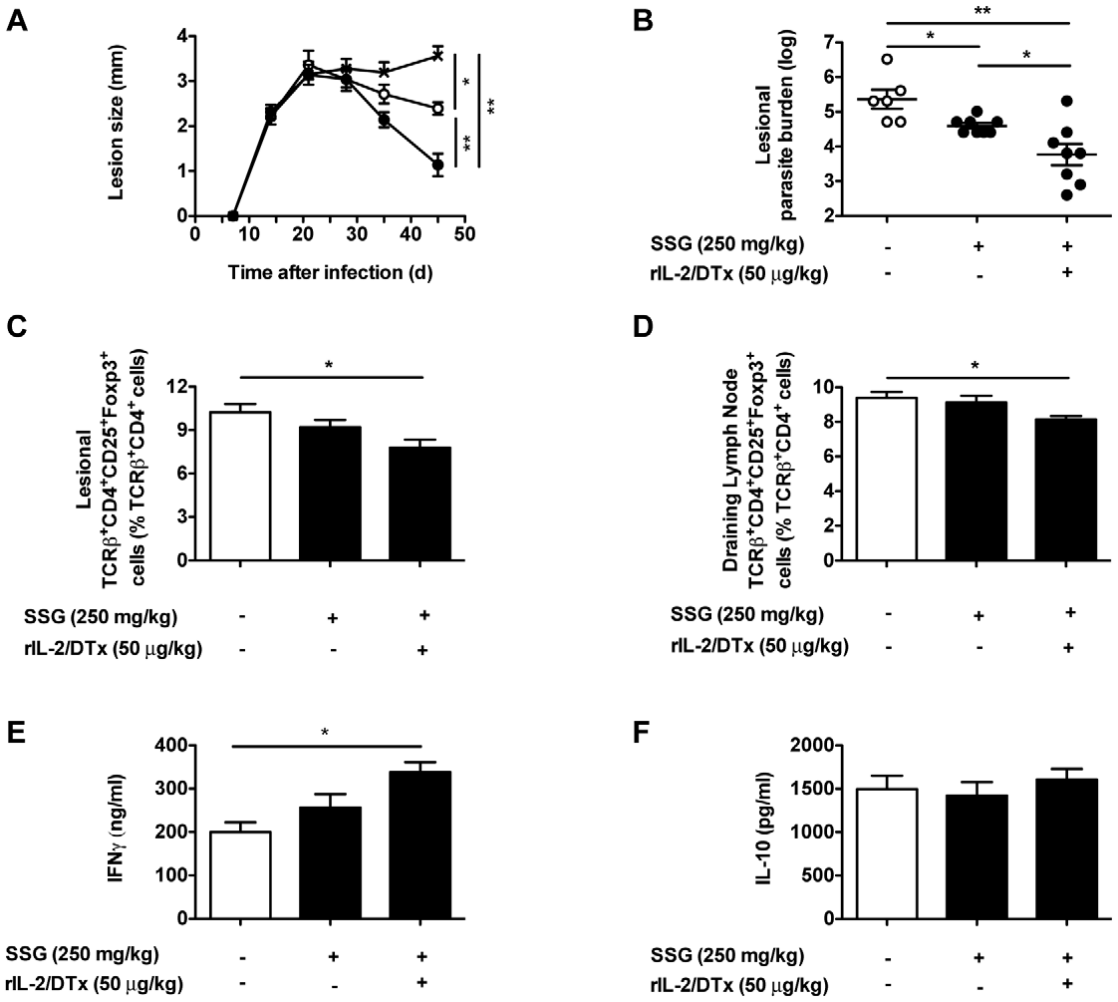
Enhanced resolution of infection with combined therapy correlates with amplification of the effector immune response. C57BL/6 mice were infected as in Figure 2; treatment was begun 30 d after infection. (**A**) Lesion size. Mice were treated: (1) daily for 10 d with SSG (250 mg/kg; open circles); (2) daily for 10 d with SSG (250 mg/kg) plus three times at 5 d intervals with rIL-2/DTx (50 µg/kg; filled circles); or (3) with normal saline (per route and schedule for 10 d SSG plus 3 doses of rIL-2/DTx; crossed symbols); harvested 45 d after infection. (**B**) Lesional parasite burden; (**C**) Lesional T_reg_ percentage; (**D**) T_reg_ percentage in draining lymph nodes; Antigen-specific (**E**) IFN-γ and (**F**) IL-10 secretion by leukocytes isolated from lymph nodes draining lesional sites and cultured in presence of soluble *Leishmania* antigen (quantified by ELISA); Data represent means +/− SE of 6–8 animals/group in a single experiment (with individual data points shown for parasite burden); (**A**) MANOVA *P*<0.05; (**A–F**) statistical analysis on data obtained 45 d after infection; ANOVA *P*<0.05; Tukey's correction; *P<0.05, **P<0.001.

Based on this, we examined the effects of the addition of rIL-2/DTx to standard SSG therapy on a range of immune parameters important in controlling the course of experimental infection with *L. major*. Mice were treated with SSG, rIL-2/DTx and/or vehicle controls beginning 30 days after infection. In concert with significant effects on lesion size and lesional parasite burden (Figure 4A and B), the addition of rIL-2/DTx to SSG led to a significant reduction in T_reg_ in lesions and draining lymph nodes (Figure 4C and D). Combined therapy with rIL-2/DTx and SSG also led to a significant increase in antigen-specific IFN-γ production by cells isolated from draining lymph nodes, compared with control or SSG treatment alone (Figure 4E). No differences in antigen-specific IL-10 production were observed (Figure 4F).

**Figure 5:**
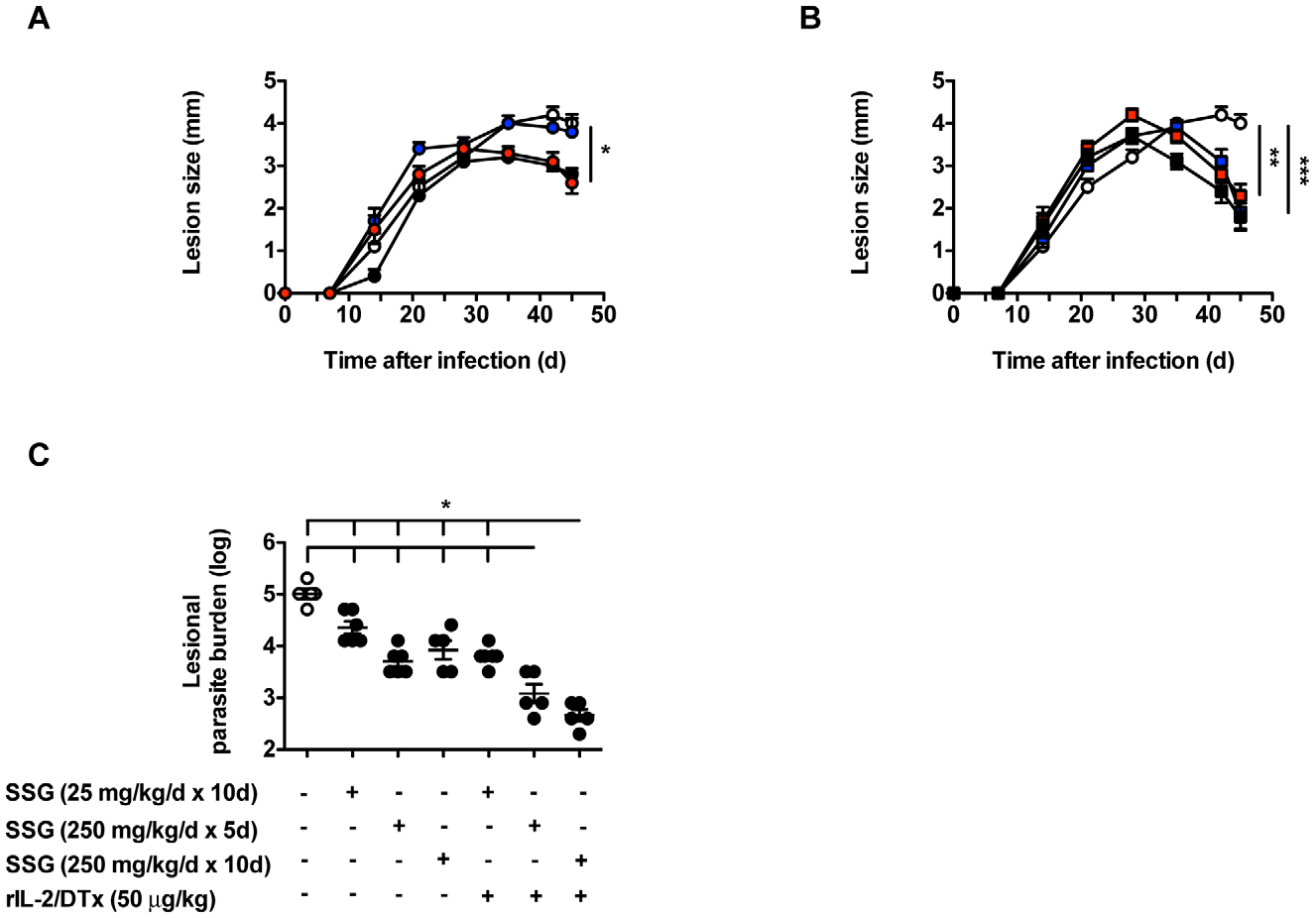
Addition of rIL-2/DTx to antibiotic therapy allows for treatment duration reduction in experimental cutaneous leishmaniasis. C57BL/6 mice were infected as in Figure 2. (**A** and **B**) lesion size. Beginning 30 d after infection, mice were treated as follows: (1) SSG 250 mg/kg daily for 10 d [black circles]; (2) SSG 25 mg/kg daily for 10 d [blue circles]; (3) SSG 250 mg/kg daily for 5 d [red circles]; (4) SSG 250 mg/kg daily for 10 d+rIL-2/DTx 50 µg/kg 3 times at 5 d intervals [black squares]; (5) SSG 25 mg/kg daily for 10 d+rIL-2/DTx 50 µg/kg 3 times at 5 d intervals [blue squares]; (6) SSG 250 mg/kg daily for 5 d+rIL-2/DTx 50 µg/kg 3 times at 5 d intervals [red squares]; (7) normal saline (per route and schedule for 10 d SSG plus 3 doses of rIL-2/DTx) [open circles]. Note, while regimens are separated into 2 panels for ease of visibility, the control (saline) group in both panels is one and the same. (**A** and **B**) MANOVA *P*<0.0004; ANOVA *P*<0.001; Tukey's correction; *P<0.05, **P<0.01, ***P<0.001. Linear random effects modeling, done in a second order of analysis, rejected the null hypothesis of all treatments having equal effects (*P*<0.001), and sorted the therapeutic efficacy of the regimens as follows, from best to worst: groups 4 and 6; groups 1, 3 and 5; groups 2 and 7. (**C**) Lesional parasite burden quantified in the indicated groups, 45 d after infection. ANOVA *P*<0.001; Tukey's correction; *P<0.05. Data represent means +/− SE of 5–6 mice/group in a single experiment (with individual data points shown for parasite burden).

We subsequently examined whether the addition of rIL-2/DTx allowed for a reduction in SSG dose or duration. Notably, as shown in Figure 5, the added clinical benefit—reduction in lesion size and parasite burden—afforded by adjunct therapy with rIL-2/DTx allowed for at least a halving of the duration of SSG treatment needed: in terms of both lesion size and parasite burden, rIL-2/DTx+5d of SSG was more effective than the full 10d regimen of SSG alone. Such combination therapy with rIL-2/DTx also allowed for SSG dose sparing: rIL-2/DTx+SSG 25 mg/kg/d for 10d had equivalent effects on lesion size and parasite burden as SSG 250 mg/kg/d for 10d alone (Fig. 5).

### Combined therapy with rIL-2/DTx and sodium stibogluconate also exhibits additive efficacy in genetically susceptible mice

**Figure 6:**
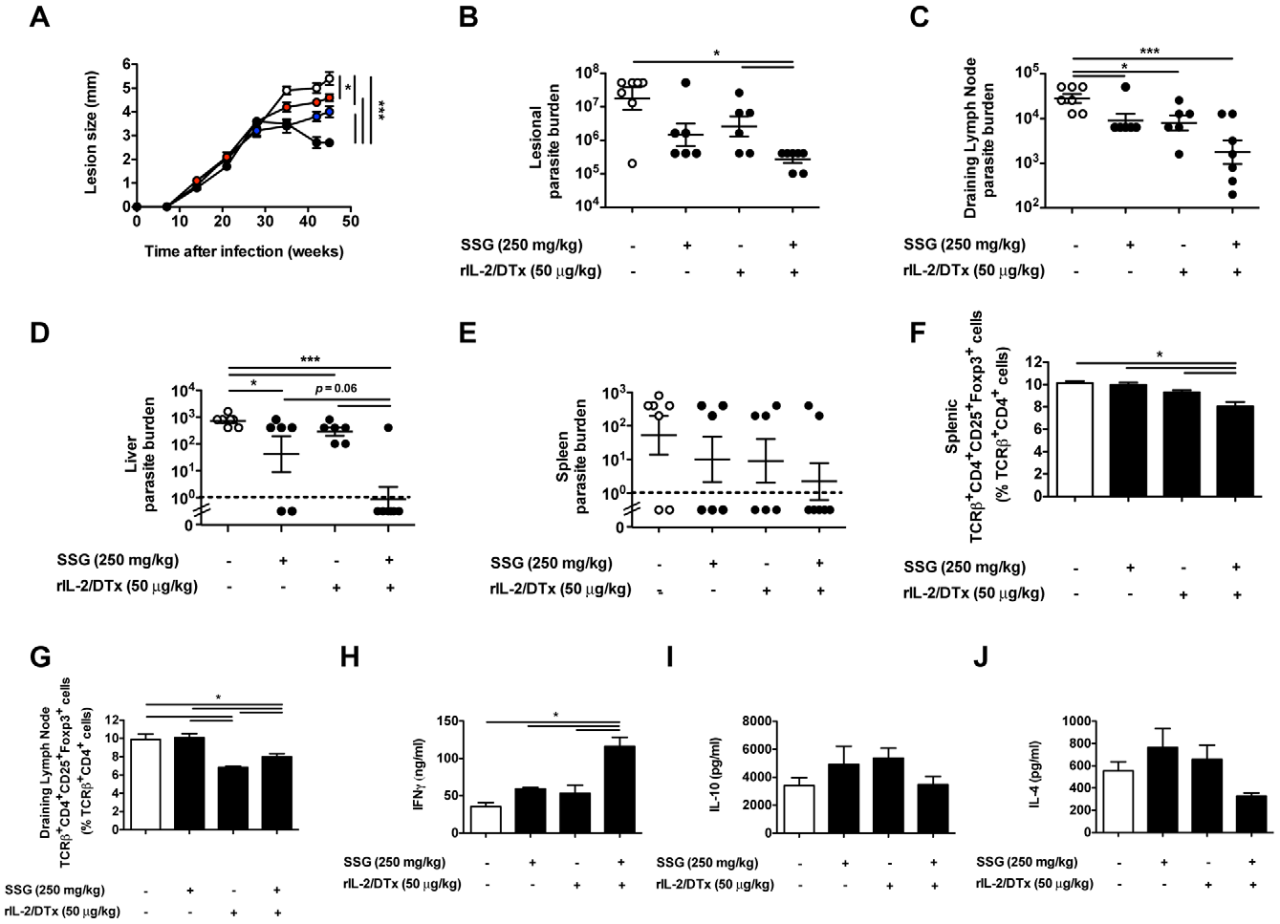
Combined therapy enhances resolution of experimental *L. major* infection in BALB/c mice. BALB/c mice were infected as in Figure 2. (**A**) Lesion size. Beginning 30 d after infection, mice were treated: daily for 10 d with SSG (250 mg/kg; blue circles); 3 times at 5 d intervals with rIL-2/DTx (50 µg/kg; red circles); daily for 10 d with SSG (250 mg/kg) plus three times at 5 d intervals with rIL-2/DTx (50 µg/kg; black circles); or with normal saline (per route and schedule for 10 d SSG plus 3 doses of rIL-2/DTx; open circles). (**B**) Lesional parasite burden; (**C**) Parasite burden in draining lymph nodes; (**D**) Parasite burden in liver (dotted line represents the limit of detection in the assay); (**E**) Parasite burden in spleen; (**F**) Splenic T_reg_ (TCRβ^+^CD4^+^CD25^+^Foxp3^+^) percentage; (**G**) Draining lymph node T_reg_ percentage; and antigen-specific (**H**) IFN-γ, (**I**) IL-10 and (**J**) IL-4 secretion (by leukocytes isolated from lesional lymph nodes and cultured in presence of soluble *Leishmania* antigen) were quantified 45 d after infection. Data represent means +/− SE of 6–7 mice/group in a single experiment (with individual data points shown for parasite burden); (**A**) MANOVA *P*<0.0003; ANOVA *P*<0.0001; Tukey's correction; *P<0.05, **P<0.01, ***P<0.001. (**B–E**) Non-parametric ANOVA (Kruskal-Wallis test for over-all comparison of treatment to no treatment); (**B**) *P*<0.02; (**C**) *P*<0.006; (**D**) not statistically significant; (**E**) *P*<0.005. Wilcoxon test (for pairwise comparisons), (**B–E**) *P<0.05, **P<0.01. (**F–H**) ANOVA *P*<0.0001; Tukey's correction; *P<0.05, **P<0.01, ***P<0.001.

To define the effect of rIL-2/DTx therapy on leishmaniasis in the face of genetic susceptibility, we turned to BALB/c mice, mice that fail to heal *L. major* infection. Of interest, additive therapeutic and biological efficacy was seen even in this highly susceptible strain. Whereas short-term therapy with either SSG or rIL-2/DTx restrained lesion expansion, short-term combined therapy led to a significant reduction in lesion size (Fig. 6A). Such combined therapy also led to: (i) a significant reduction in parasite burden in lesions, draining lymph nodes, and liver (along with a trend towards a decrease in splenic parasite burden) (Fig. 6 B–E); (ii) modest if significant suppression of T_reg_ in draining lymph nodes and spleen (Fig. 6 F and G); and (iii) significant augmentation antigen-specific IFN-γ production (in the absence of significant effects on IL-10 and IL-4 production) by cells isolated from draining lymph nodes (Fig. 6 H–J).

## Discussion

Considerable data suggest likely benefit for immunomodulatory approaches to therapy in leishmaniasis, a neglected tropical infection that continues to cause a great burden of morbidity and mortality in the tropics. Our data confirm that rIL-2/DTx administration leads to transient depletion of TCRβ^+^CD4^+^CD25^+^Foxp3^+^ T_reg_, demonstrating that this depletion is limited by development of antibodies to DTx after multiple doses. Our data further suggest potential therapeutic promise for rIL-2/DTx in cutaneous leishmaniasis. rIL-2/DTx-mediated T_reg_ suppression was associated with increased antigen-specific IFN-γ production, enhanced lesion resolution and decreased parasite burden during experimental *L. major* infection (in the absence of any obvious qualitative differences in lesion histology [data not shown]). Combined administration of rIL-2/DTx and sodium stibogluconate had additive therapeutic effects, allowing for a shortening of the needed duration or dose of SSG therapy. It should be remarked that, whereas these data have potential therapeutic implications for human cutaneous leishmaniasis, the implications are less clear for visceral leishmaniasis. T_reg_ have not been definitively been implicated as providing a key source of immune counter-regulation in either experimental or human visceral leishmaniasis, settings in which it is likely that IL-10 production by other T cells is more important [19], [24], [44].

The ability of rIL-2/DTx to deplete T_reg_ has been somewhat controversial. Studies in humans and African green monkeys that quantified T_reg_ by analyzing Foxp3 mRNA expression in peripheral blood CD4^+^ T cells or bulk peripheral blood mononuclear cells, or via enumeration of CD25-expressing CD4^+^ T cells within peripheral blood, suggested that rIL-2/DTx administration failed to significantly deplete T_reg_ [37], [45], [46]. However, direct flow cytometric quantification of Foxp3-expressing CD4^+^ T cells has indicated that rIL-2/DTx treatment leads to T_reg_ depletion in humans [30], [31], [43], something that has been replicated in mouse models [33], [34]. Our data provide insight into the kinetics of such treatment. A single dose of rIL-2/DTx leads to significant depletion of splenic T_reg_ in mice, although such depletion is reversed as early as two weeks after rIL-2/DTx administration. Further, these data show that repetitive administration of rIL-2/DTx leads to sustained reduction of splenic T_reg_ for up to four weeks of treatment in mice, something observed in humans as well [30], [45]. However, long-term repetitive administration of rIL-2/DTx appears to be limited by the generation of antibodies to DTx. This indicates that the efficacy of rIL-2/DTx immunomodulation is likely to be temporally limited. This narrow temporal window, along with the partial depletion of T_reg_ numbers achieved, may actually be beneficial in limiting potential deleterious over-activation of immune responses with sustained T_reg_ depletion.

Monoclonal antibody-mediated depletion of CD25-expressing cells has been reported to facilitate parasite eradication in experimental leishmaniasis [25], [26], [27], [28]. Further, it should be noted that, prior to recognition of regulatory T cells, IL-2 was shown to be necessary for disease progression during experimental *L. major* infection of susceptible murine hosts [25], [47], [48] (although the effects of IL-2 manipulation on the course of experimental infection with *L. donovani* appears to be more complicated, suggesting the need for caution in extrapolating these findings to visceral leishmaniasis [49], [50]). While the effects of IL-2 on *L. major* infection have remained mechanistically undefined, one of the principal non-redundant functions of IL-2 appears to be regulation of T_reg_ numbers [51]. Our finding that rIL-2/DTx-mediated blunting of T_reg_ numbers is associated with increased immune-mediated clearance of *L. major* is thus not unexpected. It is acknowledged that the mechanism underlying the beneficial effects of rIL-2/DTx therapy in experimental leishmaniasis remains mechanistically under-defined. In particular, it may well be that rIL-2/DTx administration results, as well, either directly or indirectly, in biologically important changes in other cellular subsets that modulate anti-leishmanial immune responses. Of note in this regard, long-term depletion of CD25-expressing cells with declizumab has been shown to increase regulatory NK cell numbers, as well as decrease T_reg_ numbers, in humans with multiple sclerosis— with regulatory NK cell changes correlating with disease suppression in this autoimmune disease [52], [53]. Further, the fate of IL-10 producing Th_1_ cells [20], [23], [54] following rIL-2/DTx administration remains unclear. However, the lack of significant differences in antigen-driven IL-10 production following *in vitro* re-stimulation suggests that rIL-2/DTx administration may not directly alter the function or the numbers of such cells.

There are many possible ways to therapeutically target T_reg_ numbers and/or function, including direct targeting through CD25, blockade of IL-10, inhibition of CTLA-4 or TGF-β, engagement of GITR, and/or activation of dendritic cells (e.g., through LPS or CD40) [55]. The first two of these methods have already shown clear efficacy in mouse models of cutaneous leishmaniasis [14], [17]. There are also, of course, theoretical reasons for caution: therapeutic targeting of T_reg_ has the potential for promoting the development or expression of autoimmune disease in susceptible hosts, and for upregulating potentially deleterious immune responses to the infecting pathogen or to co-infecting pathogens. Although there are similar concerns with other immunological approaches, these considerations suggest that, for safety, T_reg_ targeting should be as narrow as possible. Thus, if IL-10 blockade and CD25^+^ T cell targeting are both efficacious, the latter would be preferable as IL-10 is produced by many cells other than T_reg_. Similarly, while sustained targeting of T_reg_ alone eradicates *L. major* in mouse models, brief targeting of T_reg_, along with antimicrobial therapy would likely be preferable. There may also be benefit to T_reg_ targeting in concert with therapeutic vaccination. It should also be noted that the use of biologicals to inhibit immunological pathways (e.g., cytokine inhibition) has, in general, been easier and fraught with fewer side effects than the use of biologicals to activate immunological pathways (e.g., cytokine therapy). Thus, inhibition of inhibitory pathways (e.g., targeting of CD25^+^) cells may be preferable to direct immune stimulation (e.g., of dendritic cells). Together, these considerations suggest practical therapeutic utility for direct targeting of CD25^+^ cells in leishmaniasis and other chronic infections in which T_reg_ play an important biological role in hindering host-mediated immune clearance. More broadly, the current data suggest that rational reinvestigation of the efficacy of drugs approved for other indications in experimental models of neglected tropical diseases has promise in providing needed new candidates to the drug discovery pipeline.

## Supporting Information

Figure S1 **Gating strategy for T_reg_ quantification.** Live cells in the TCRβ^+^CD4^+^ gate were analyzed for CD25 and Foxp3 expression as indicated.
(TIF)
