# Supplementary figures and images for: Therapeutic Enhancement of Protective Immunity during Experimental Leishmaniasis
Source: PLoS Negl Trop Dis. 2011 Sep 6;5(9):e1316. doi: 10.1371/journal.pntd.0001316 (PMC3167777; doi:10.1371/journal.pntd.0001316)

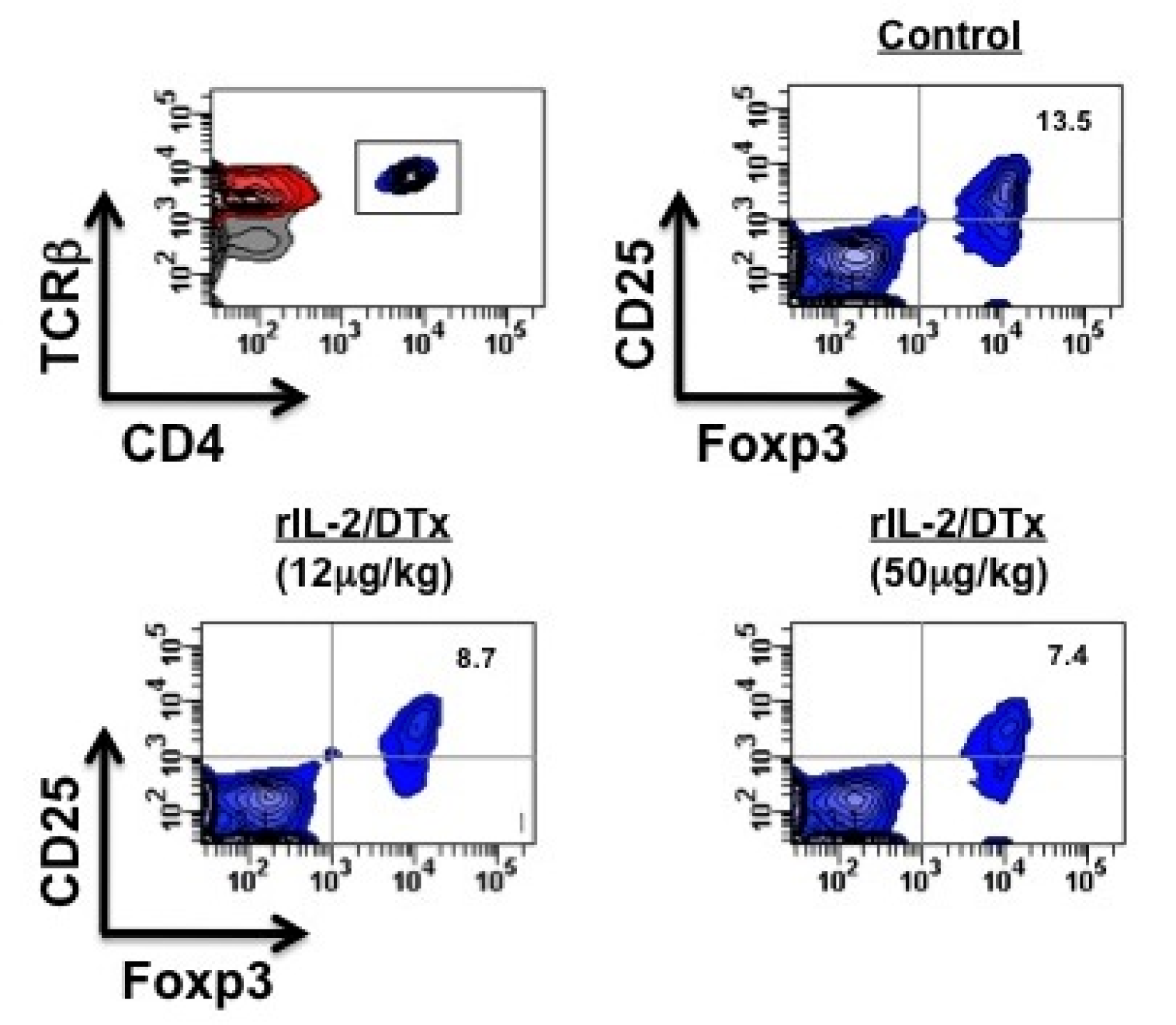

Supplement: Figure S1 — Gating strategy for Treg quantification. Live cells in the TCRβ+CD4+ gate were analyzed for CD25 and Foxp3 expression as indicated. (TIF) [file pntd.0001316.s001.tif]
